# Risk factors for intensive care unit admission or mortality in adults hospitalized for COVID-19 at high altitude

**DOI:** 10.17843/rpmesp.2022.392.10721

**Published:** 2022-06-30

**Authors:** Niza Alva, Giovana Asqui, Germán F. Alvarado, Francisco Muchica

**Affiliations:** 1 Programa de Medicina, Universidad Peruana de Ciencias Aplicadas, Lima, Peru Universidad Peruana de Ciencias Aplicadas Programa de Medicina Universidad Peruana de Ciencias Aplicadas Lima Peru; 2 Hospital Regional Manuel Núñez Butron, Puno, Peru. Hospital Regional Manuel Núñez Butron Puno Peru

**Keywords:** COVID-19, Mortality, Inpatients, Altitude, Oximetry

## Abstract

**Objective.:**

To determine the risk factors for admission to the intensive care unit (ICU) or mortality in patients hospitalized for COVID-19 in a hospital in Puno, Peru.

**Materials and methods.:**

Retrospective cohort study in adults hospitalized between April and December 2020. We evaluated Sociodemographic characteristics, vital functions, comorbidities, treatment received and its association with admission to ICU or mortality (adverse outcome). Poisson regression with robust variance was used to calculate crude and adjusted relative risks (RR) with their 95% confidence intervals (95%CI).

**Results.:**

A total of 348 medical records were analyzed. The median age in years was 42.5 (IQR: 30.0; 58.0); 38.2% were male, and 35.3% died or were admitted to the ICU. Those admitted with an oxygen saturation ≤ 75% were 2.79 times more likely to have the adverse outcome (p < 0.001), compared to those admitted with a saturation ≥ 85%; those admitted with a value between 75-79% were 2.92 times more likely to have the adverse outcome (p < 0.001); likewise, those admitted with saturation between 80-84% were 1.70 times more likely to have the adverse outcome; however, the difference was not statistically significant (p=0.066). In addition, male patients, RR= 1.75 (p<0.001); those aged > 40 years, RR 3.5 (p=0.001); those with tachypnea, RR=1.66 (p=0.010); or with diabetes, RR = 1.53 (p=0.011) had higher risk of presenting the adverse outcome.

**Conclusions.:**

The risk factors for ICU admission or mortality due to COVID-19 were male sex, age over 40 years, low saturation, diabetes and tachypnea.

## INTRODUCTION

A significant number of severe pneumonia cases were reported in Wuhan, China in December 2019 ^1^. Subsequently, this new disease would be identified as COVID-19, caused by severe acute respiratory syndrome type 2 virus (SARS-CoV-2) ^1^. On March 11, 2020, The World Health Organization (WHO) proclaimed COVID-19 as a pandemic due to its extensive worldwide spread, suggesting that countries follow a comprehensive strategy aimed at preventing infections and deaths ^2^. As of October 26, 2021, more than 4.9 million deaths caused by this disease had been reported worldwide. In response, the Peruvian Ministry of Health (MINSA) decreed a state of national health emergency ^3^. Confirmed cases of coronavirus in the country totaled 2,197,052, with 200,118 deaths and a case fatality rate of 9.11% ^4^. By August 2020, Peru became the country with the highest COVID-19 mortality per number of inhabitants ^4^ ^,^ ^5^.

There are studies that suggest that the prevalence and impact of COVID-19 could be diminished by a potential protective factor such as altitude ^6^ ^,^ ^7^; either due to the drastic changes that usually exist in these environments, high ultraviolet radiation and dryness of the air ^6^. Likewise, the decreased expression of angiotensin-converting enzyme (ACE) in the pulmonary endothelium of high-altitude inhabitants could generate a physiological protective factor as it is the binding site of SARS-CoV-1 and SARS-CoV-2 ^6^ ^,^ ^7^. However, these are ecological studies, with inconclusive statements, and more research is needed.

There are several factors associated with mortality due to this disease, which can be grouped into sociodemographic characteristics, vital functions, comorbidities, ancillary examinations, time of illness and treatment received ^3^. Among the sociodemographic characteristics, male sex and age over 60 years stand out ^3^ ^,^ ^8^ ^-^ ^10^. Obesity (BMI > 30) ^3^ ^,^ ^11^ ^,^ ^12^, diabetes *mellitus* ^11^ ^-^ ^14^ and arterial hypertension (AHT) ^3^ ^,^ ^11^ ^,^ ^15^ are the most important the comorbidities. These characteristics predispose any individual to a higher risk of SARS-CoV-2 infection or more severe disease.

Oxygen saturation (SatO^2^) is an important prognostic factor, since having a low percentage at hospital admission is associated with higher mortality ^3^ ^,^ ^15^ ^,^ ^16^. A study conducted in Lima, a city at an altitude of 161 m, found that having saturation < 90% on admission was associated with a higher risk of death compared to those who arrived with > 90% ^3^. We have not found studies that determine what percentage saturation indicates a poor prognosis in high-altitude residents. However, it is known that high-altitude residents have different SatO_2_ values than residents at sea level. A study conducted in healthy residents of Huánuco (1894 m altitude) and Cerro de Pasco (3399 m altitude) found that the mean saturation was 96.2 and 87.0%, respectively, concluding that the higher the altitude of residence, the more noticeable the changes in the normal values of SatO_2_ ^17^, it should be noted that this study was conducted outside the context of a pandemic.

Puno is a region located at an altitude of 3827 m, so the results of this study may be useful to understand the dynamics of the disease in high-altitude populations and to propose future studies.

This research aimed to determine the risk factors for intensive care unit (ICU) admission or mortality in a high-altitude population of adults hospitalized for COVID-19 in a hospital in Puno, Peru.

**KEY MESSAGES**

Motivation for the study: Risk factors for adverse outcome due to COVID-19 are not well defined in populations in altitudes greater than 3000 m.

Main findings: Factors associated with death due to COVID-19 were age > 40 years, male sex, diabetes, admission with oxygen saturation less than 80% and respiratory rate > 22 bpm.

Implications: Identifying these risk factors in a population located at 3827 m altitude will allow us to recognize patients with worse prognosis in order to implement adequate measures.

## MATERIALS AND METHODS

### Design and context

A retrospective cohort study was designed at the Manuel Núñez Butrón Regional Hospital (HRMNB) in Puno, Peru, that included adult patients hospitalized in the COVID area between April and December 2020. The HRMNB is a category II hospital, administered by the Ministry of Health (MINSA) located at an altitude of 3827 m. The first COVID-19 cases were recorded in early April, with the first wave occurring between July and August.

### Population

We included adult patients (≥18 years), hospitalized in COVID areas of the HRMNB who previously resided in Puno for at least two months. Patients who voluntarily withdrew and those without confirmed diagnosis by a rapid test were excluded.

### Sample

We carried out a census of patients hospitalized in the COVID area of the HRMNB of Puno from April to December 2020, obtaining 542 medical records. Epidat 4.2 was used to define a minimum sample size. Based on a previous study ^3^, we constructed a contingency table and calculated the probability of dying between exposed and unexposed; we considered a ratio of 0.33 with a confidence level of 99.9% and power of 99.9%. p1 represents the probability of dying in patients with low saturation (<85%) being 61.6%; while; p2, is the probability of dying in patients without low saturation (≥ 85%) which was 14%. The minimum required sample size was 192 and considering 10% of incorrectly filled histories, it was 214.

### Variables

The dependent variable was the adverse outcome, which consisted of admission to the ICU or in-hospital mortality. This variable was categorical dichotomous (died or was admitted to the ICU / did not die or was not admitted to the ICU).

Independent variables were age in years (<40 / 40-59 / ≥60), sex (female / male), time of illness (days), oxygen saturation on admission (≤75% / 76% - 79% / 81% - 84% / ≥85%), respiratory rate (tachypnea > 22 bpm / normal), among other vital functions. Comorbidities had dichotomous responses (yes / no) and include diabetes *mellitus*, AHT, chronic kidney disease, congestive heart failure and obesity. Regarding laboratory tests, hemoglobin (g/dL) and glucose (mg/dL) are shown as numerical variables, in addition to lymphopenia (≤1400 U/mm^3^ / >1400 U/mm^3^) and blood type (O+, A+, B+). Medications received during hospitalization had dichotomous response (yes/no) and included dexamethasone, azithromycin, hydroxychloroquine and ivermectin, among others. It should be noted that the vital functions and laboratory test values we considered were those obtained at hospital admission.

### Data Collection

Data collection was carried out after approval from the Research Ethics Committee of the Universidad Peruana de Ciencias Aplicadas and authorization from the Regional Health Directorate (DIRESA) of Puno. We obtained access to the medical records of patients hospitalized by COVID-19 at the HRMNB of Puno between April and December 2020. Those that met the selection criteria were selected. Between June and July 2021, the data collection form was filled out with the variables chosen by literature review, then the data were organized in Excel.

### Data analysis

We used the STATA v16 statistical analysis program. For the univariate analysis we calculated percentages for categorical variables, measures of central tendency and dispersion for quantitative variables, the mean and standard deviation (SD) were calculated if there was normality, otherwise, median and interquartile range (IQR). For the bivariate analysis, we used Student’s t test / Mann Whitney U test and chi-square / Fisher’s test, according to compliance with assumptions. Finally, for the multivariate analysis, we used Poisson regression with robust variance to calculate the crude and adjusted relative risk (RR) with 95% confidence intervals (95%CI). An alpha of 0.05 was considered. Variables were entered into the model according to epidemiological/theoretical criteria. Collinearity with VIF (variance inflation factor) was assessed using a cutoff point of 2.

### Ethical aspects

The protocol was approved by the Ethics Committee of the Universidad Peruana de Ciencias Aplicadas (SCEI 024-01-22 PI 496-20) and authorized by the DIRESA of Puno. Since the data were already in the medical records, the research team had no direct contact with the research subjects and confidentiality was maintained. PRISA registration code: EI00001585.

## RESULTS

**Figure 1:**
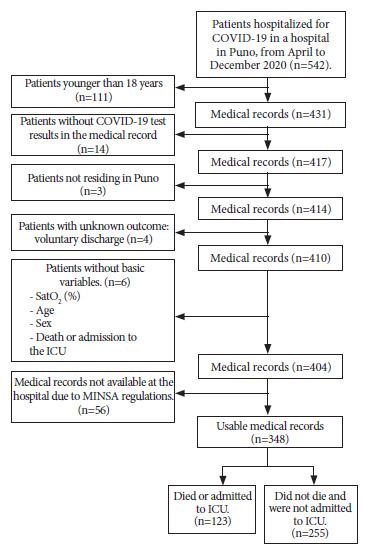
Flowchart of the selection process of the medical records of patients hospitalized by COVID-19 in a hospital in Puno, Peru.

A total of 542 medical records were obtained from patients hospitalized due to COVID-19 in the hospital of Puno, from April to December 2020. Of these, 194 were excluded because they did not meet inclusion criteria or because they were not available. Finally, 348 were included as part of the analyzed sample (Figure 1).

### Descriptive analysis

With respect to the baseline characteristics (Table 1), 48.2% were under 40 years of age, 29.1% were between 40 and 59 years of age and 22.7% were 60 years of age or older; 61.8% were female. Regarding the outcome, 18.4% were admitted to the ICU, while 5.5% used invasive mechanical ventilation, and in-hospital mortality was 29.9%. In terms of comorbidities, 13.8% had diabetes, 7.5% had AHT, 4.6% had obesity and, finally, 21.8% had one comorbidity and 6.4% had two or more of them.

**Table 1:** Characteristics of patients hospitalized in COVID areas. Manuel Núñez Butrón Hospital. Puno, Peru 2020.

Variables	n (%)
Basic characteristics	
Age (years)	
<40	168 (48.2)
40 - 59	101 (29.1)
≥ 60	79 (22.7)
Sex	
Male	133 (38.2)
Female	215 (61.8)
Reason for admission	
COVID	157 (45.1)
Other	191 (54.9)
Outcome	
Admission to ICU	64 (18.4)
Mechanical ventilation	19 (5.5)
Hospital mortality	104 (29.9)
Hospital stay (days) ^a^	3.8 (1;5)
Comorbidities	
Diabetes	48 (13.8)
AHT	26 (7.5)
Asthma	1 (0.3)
Tuberculosis	1 (0.3)
Cancer	1 (0.3)
CKD	5 (1.4)
CHF	6 (1.7)
COPD	4 (1.2)
Interstitial lung disease	2 (0.6)
VIH	1 (0.3)
Obesity	16 (4.6)
Number of comorbidities	
0	247 (71.8)
1	75 (21.8)
≥ 2	22 (6.4)
Vital functions	
Oxygen saturation (%)	
≥ 85	242 (69.9)
80 - 84	19 (5.5)
76 - 79	21 (6.1)
≤ 75	64 (18.5)
Temperature (°C) ^a^	36.6 (36.2;36.8)
Respiratory rate (bpm)	
Normal	163 (48.7)
Tachypnea	172 (51.3)
Heart rate (bpm) ^a^	89.9 (78;100)
Systolic blood pressure (mmHg) ^a^	117.1 (105;128)
Diastolic blood pressure (mmHg) ^a^	74.5 (66;83)
Diagnostic	
Clinical diagnostic	168 (48.3)
Radiologic diagnostic	
Positive	77 (22.6)
Negative	5 (1.6)
Not performed	250 (75.8)
Rapid test	
Positive IgM rapid test	233 (66.9)
Positive IgG rapid test	285 (81.9)
Molecular test	
Positive	14 (4.2)
Negative	0 (0.0)
Not performed	321 (95.8)
Management	
Anticoagulants	
Full dose	51 (14.6)
Prophylactic dose	57 (16.4)
Did not receive	240 (69.0)
Non-invasive ventilation	
Binasal cannula	75 (21.6)
Reservoir mask	73 (29.9)
Medications received	
Dexamethasone	130 (37.4)
Methylprednisolone	2 (0.6)
Prednisone	1 (0.3)
Acetaminophen	20 (5.8)
Ibuprofen	5 (1.4)
Metamizole	151 (43.4)
Tramadol	49 (14.1)
Azithromycin	81 (23.3)
Meropenem	28 (8.1)
Imipenem	41 (11.8)
Vancomycin	96 (27.6)
Cefepime	1 (0.3)
Ceftriaxone	102 (29.3)
Cefazoline	7 (2)
Ampicillin	26 (7.3)
Amoxicillin	7 (2.01)
Clindamycin	30 (8.62)
Gentamicin	23 (6.6)
Ciprofloxacin	12 (3.5)
Metronidazole	5 (1.4)
Hydroxychloroquine	83 (23.9)
Ivermectin	78 (22.4)
ACEI	20 (5.8)
Vasopressors	14 (4)
Omeprazole	152 (43.7)
Salbutamol	69 (19.8)
Ipratropium bromide	68 (19.5)

a median (interquartile range)

Some values may have different totals due to missing data.

ICU: intensive care unit. AHT: arterial hypertension. CKD: chronic kidney disease. CHF: congestive heart failure. COPD: chronic obstructive pulmonary disease. HIV - acquired immunodeficiency virus. GOT - glutamic oxaloacetic transaminase. GPT - glutamic-pyruvic transaminase. CRP - C-reactive protein. LDH - lactate dehydrogenase.

Regarding oxygen saturation, 69.9% of patients were admitted with a saturation greater than 85%, 5.5% were admitted with a saturation ranging from 80 to 84%, 6.1% with a saturation ranging from 76 to 79% and 18.5% were admitted with less than 75% saturation.

Regarding diagnosis, 48.3% had clinical diagnosis. In addition, 66.9% were positive for the IgM rapid test and 81.9% were positive for the IgG rapid test. As for laboratory tests, the mean hemoglobin was 15.1 g/dL with a standard deviation (SD) of 3.0. In addition, 46.7% had lymphopenia and 33.9% had leukocytosis. The median glucose was 102 mg/dL (IQR: 84.1;124.0). In terms of management, in noninvasive ventilation, i.e. ventilatory support without endotracheal intubation, 29.9% received a reservoir mask; meanwhile, 21.6% of patients received binasal cannula. With respect to the treatment, 37.4% received dexamethasone. On the other hand, 23.3% received azithromycin, 23.9% took hydroxychloroquine and 22.4% took ivermectin.

### Bivariate analysis

During the bivariate analysis, we observed a statistically significant association between age and adverse outcome (p < 0.001) as well as with male sex (p < 0.001) and reason for admission (p < 0.001) (Table 2).

**Table 2:** Factors associated with admission to the ICU or death. Bivariate analysis. Manuel Núñez Butrón Hospital. Puno, Peru 2020.

Variables	Died or admitted to ICU n (%)	Did not die and was not admitted to ICU n (%)	p-value
Basic characteristics			
Age (years)^ a^			
< 40	9 (5.4)	159 (94.6)	
40 - 59	57 (56.4)	44 (43.6)	<0.001
≥ 60	57 (72.2)	22 (27.8)	
Sex ^a^			
Male	88 (66.2)	45 (33.8)	<0.001
Female	35 (16.3)	180 (83.7)	
Reason for admission ^a^			
Other	18 (9.4)	173 (90.6)	<0.001
COVID	105 (66.9)	52 (33.1)	
Hospital characteristics			
Hospital stay (days) ^b^	4.4 (2.0;6.0)	3.4 (1.0;4.0)	0.022
Comorbidities			
Diabetes ^a^			
No	86 (28.7)	214 (71.3)	<0.001
Yes	37 (77.1)	11 (22.9)	
AHT ^a^			
No	110 (34.2)	212 (65.8)	0.104
Yes	13 (50.0)	13 (50.0)	
CKD ^c^			
No	119 (34.7)	224 (65.3)	0.055
Yes	4 (80.0)	1 (20.0)	
CHF ^c^			
No	118 (34.5)	224 (65.5)	0.022
Yes	5 (83.3)	1 (16.7)	
Obesity ^a^			
No	109 (32.8)	223 (67.2)	<0.001
Yes	14 (87.5)	2 (12.5)	
Vital functions			
Saturation (%)			
≥ 85	29 (11.9)	213 (88.1)	
80 - 84	11 (57.9)	8 (42.1)	<0.001
75 - 79	19 (90.5)	2 (9.5)	
≤ 75	62 (96.9)	3 (3.1)	
Respiratory rate ^a^			
Normal	17 (10.4)	146 (89.6)	<0.001
Tachypnea	98 (57.0)	74 (43.0)	
SBP (mmHg) ^b^	111 (95.0;130.0)	120 (109.0;127.0)	0.017
DBP (mmHg) ^b^	73 (60.0;81.0)	75 (69.5;83.0)	0.025
Management			
Anticoagulants ^a^			
Full dose	38 (74.5)	13 (25.5)	<0.001
Prophylactic dose	35 (61.4)	22 (38.6)	
No	50 (20.8)	190 (79.2)	
Dexamethasone ^a^			
No	40 (18.4)	178 (81.6)	<0.001
Yes	83 (73.8)	47 (36.2)	
Azithromycin ^a^			
No	68 (25.5)	199 (74.5)	<0.001
Yes	55 (67.9)	26 (32.1)	
Hydroxychloroquine ^a^			
No	67 (25.3)	198 (74.7)	<0.001
Yes	56 (67.5)	27 (32.5)	
Ivermectin ^a^			
No	70 (25.9)	200 (77.1)	<0.001
Yes	53 (67.9)	25 (32.1)	

a Chi-square test.

b Median (interquartile range) Mann Whitney U test.

c Fisher’s exact test

AHT: hypertension. CKD: chronic kidney disease. CHF: congestive heart failure. SBP: systolic blood pressure. DBP: diastolic blood pressure.

Regarding comorbidities, diabetes (p<0.001) and obesity (p<0.001) had a significant association with adverse outcome; no significant association was observed with AHT (p=0.104).

As for vital signs, we found a significant association between oxygen saturation and adverse outcome (p < 0.001), as well as with respiratory frequency (p < 0.001), lower systolic blood pressure (p = 0.017) and lower diastolic blood pressure (p = 0.025).

With respect to laboratory tests, a significant association was found between adverse outcome and higher hemoglobin level (p < 0.001), as well as with higher glucose level (p < 0.001) and lymphopenia (p < 0.001). However, no significant association was found between adverse outcome and blood group (p = 0.200).

Regarding the treatment, a significant association was found between the adverse outcome and dexamethasone (p < 0.001), azithromycin (p < 0.001), hydroxychloroquine (p < 0.001) and ivermectin (p < 0.001).

### Analysis of multiple variables

During the multivariate analysis we found a statistically significant association between age and the adverse outcome, with those between 40 and 59 years of age having 3.7 times the probability of developing an adverse outcome (p < 0.001) and those over 60 years of age having 3.5 times the risk compared to those under 40 years of age (p = 0.001) (Table 3). Likewise, men had 1.8 times the risk of adverse outcome (p < 0.001) after adjusting for the rest of the variables in the equation. Regarding comorbidities, patients with diabetes had 1.3 times the risk of developing the adverse outcome (p = 0.011) and obese patients had 1.2 times the risk of developing it; however, the association was not significant (p = 0.260). On the other hand, there was no statistically significant association with AHT (p = 0.851). Patients with oxygen saturation ranging between 80 and 84% had 1.7 times the risk of developing an adverse outcome compared to those admitted with more than 85%, without a significant association (p = 0.066); on the other hand, those with values between 75 and 79% had 2.9 times the probability of developing an adverse outcome (p < 0.001), the same for those admitted with less than 75%, who had a risk of 2.8 (p < 0.001).

**Table 3:** Factors associated with admission to the ICU or death. Analysis of multiple variables. Manuel Núñez Butrón Hospital. Puno, Peru 2020.

Variables	cRR	95%CI	p-value	aRR	95%CI	p-value
Age (years)						
< 40	Reference			Reference		
40 - 59	10.53	5.44;20.36	<0.001	3.71	1.82;7.57	<0.001
≥ 60	13.46	7.02;25.82	<0.001	3.53	1.73;7.21	0.001
Sex						
Female	Reference			Reference		
Male	4.06	2.93;5.63	<0.001	1.75	1.30;2.35	<0.001
Diabetes						
No	Reference			Reference		
Yes	2.68	2.12;3.40	<0.001	1.34	1.06;1.69	0.011
AHT						
No	Reference			Reference		
Yes	1.46	0.96;2.21	0.071	0.96	0.67;1.38	0.851
CKD						
No	Reference					
Yes	2.30	1.45;3.66	<0.001	-	-	-
CHF						
No	Reference					
Yes	2.41	1.64;3.55	<0.001	-	-	-
Obesity						
No	Reference			Reference		
Yes	2.66	2.09;3.39	<0.001	1.21	0.86;1.72	0.260
Saturation						
≥ 85	Reference			Reference		
80 - 84	4.83	2.88;8.07	<0.001	1.70	0.96;3.00	0.066
75 - 79	7.61	5.29;10.97	<0.001	2.92	1.99;4.30	<0.001
≤ 75	8.08	5.72;11.41	<0.001	2.79	1.89;4.11	<0.001
Respiratory rate						
Normal	Reference			Reference		
Tachypnea (>22 bpm)	5.46	3.41;8.73	<0.001	1.66	1.12;2.46	0.010
Anticoagulants						
No	Reference			Reference		
Full dose	3.57	2.66;4.80	<0.001	1.25	0.91;1.72	0.157
Prophylactic dose	2.94	2.13;4.06	<0.001	1.04	0.80;1.36	0.730
Dexamethasone						
No	Reference			Reference		
Yes	3.47	2.55;4.73	<0.001	1.04	0.77;1.41	0.769
Azithromycin						
No	Reference			Reference		
Yes	2.66	2.06;3.43	<0.001	1.05	0.79;1.40	0.705
Hydroxychloroquine						
No	Reference			Reference		
Yes	2.63	2.06;3.44	<0.001	0.90	0.73;1.12	0.386
Ivermectin						
No	Reference					
Yes	2.62	2.03;3.37	<0.001	-	-	-

cRR: crude relative risk; aRR: adjusted relative risk; AHT: hypertension; CKD: chronic kidney disease; CHF: congestive heart failure.

Patients admitted with tachypnea (> 22 bpm) had 1.7 times the risk of an adverse outcome, with a statistically significant difference (p = 0.010). There was no association between the use of anticoagulants and the risk of having an adverse outcome (p > 0.05). Similarly, there was no association between adverse outcome and dexamethasone (p = 0.769), azithromycin (p = 0.705) and hydroxychloroquine (p = 0.386). The variables “chronic kidney disease” and “congestive heart failure” were eliminated from the adjusted model due to a significant percentage of missing values and the variable “ivermectin” was eliminated due to collinearity with hydroxychloroquine.

## DISCUSSION

The risk factors identified with admission to the ICU or death were age over 40 years, male sex, admission with oxygen saturation less than 80%, admission with respiratory rate > 22, or diabetes *mellitus*. No significant association was found between adverse outcome and obesity or hypertension. As secondary findings, 18.4% of patients were admitted to the ICU and 29.9% died; 30.1% were admitted with saturation < 85%. The mortality we found was lower (29.9%) than that reported for other hospitals, 49% ^3^ and 46.4% ^18^; however, these studies would not be comparable as they are from different contexts, characteristics and times of the pandemic in the country.

Nonetheless, our study reports characteristics and risk factors similar to those described in other cohorts ^3^ ^,^ ^13^ ^,^ ^15^ ^,^ ^19^ ^-^ ^21^. We observed an association between age over 40 years and the adverse outcome, which is also evident in other studies ^9^ ^,^ ^10^ ^,^ ^13^ ^,^ ^23^, possibly due to the increased prevalence of chronic diseases in this age group. It could also be due to vulnerability to infections, explained by immunosenescence ^9^.

In this study we observed an association between male sex and outcome. This association has been described in studies carried out in cities at sea level ^3^ ^,^ ^8^ ^,^ ^10^. It has been described that biological mechanisms, such as a decreased immune response in men versus women may be involved ^23^. It has been suggested that sex hormones, such as androgens, could play an important role in regulating proteins such as transmembrane serine protease 2 (TMPRSS2), which is involved in viral entry and spread, resulting in more severe forms of COVID-19 ^24^.

We identified that having an admission oxygen saturation < 80% was a risk factor, this value differs from the cut-off point proposed in other similar studies performed in populations at sea level ^3^ ^,^ ^15^ ^,^ ^18^ and in populations with a lower altitude than Puno ^19^. This could be explained by the low oxygen pressure, since the saturation described in patients at high altitude without any pathology is lower, compared to that of residents at sea level ^17^, so it would be expected that in patients with COVID-19, the saturation would be even lower since it is a pathology that adds hypoxemia. It is difficult to establish a cut-off point in high altitude populations. An inversely proportional relationship between oxygen saturation and altitude level has been previously described ^17^. More studies are needed to quantify this relationship and determine cut-off points.

Likewise, we found a significant association between tachypnea (>22 bpm) and the adverse outcome. This relationship is understandable because an increasing respiratory rate is the physiological response to hypoxemia and acidosis ^25^. Again, it should be noted that more studies evaluating tachypnea in high-altitude conditions are needed to determine the cutoff points for poor prognosis in this population.

Regarding comorbidities, we found an association between the adverse outcome and diabetes mellitus (DM), which is similar in other studies ^11^ ^-^ ^14^, since DM involves immunosuppression due to alterations in the immune response that include increased production of proinflammatory cytokines, which add inflammation to the patient with COVID-19 ^26^. However, no association was found between the adverse outcome and obesity, as has been found in other studies ^20^ ^,^ ^22^ where the outcome was mortality and, like DM, obesity involves a proinflammatory state due to cytokines (TNF-alpha, MCP-1 and IL-6) produced by adipose tissue ^12^. On the other hand, although studies report an association between mortality and AHT ^3^ ^,^ ^11^ ^,^ ^15^, no association was found between AHT and adverse outcome in the adjusted analysis. It has been proposed that there is a higher expression of ACE in hypertensive patients due to treatment with ACE inhibitors, which could increase the risk of severe forms of COVID-19 ^14^.

We did not find statistically significant associations in the laboratory results; however, there is literature describing that severe patients in high altitude areas show a decrease of the platelet count.

Finally, no significant association was found between adverse outcome and receiving anticoagulants (enoxaparin) either in prophylactic dose (40 mg) or full dose (60 mg), nor with dexamethasone, azithromycin or hydroxychloroquine. Regarding hydroxychloroquine, there are studies that show that there is no benefit in reducing mortality compared to those who do not receive it ^27^ ^,^ ^28^. On the contrary, there is literature that supports the association between the use of low-dose dexamethasone and a reduction in mortality in patients with severe COVID-19 ^29^.

The main limitation of this study is the use of the rapid test, due to its low diagnostic sensitivity. However, it proved to be the most accessible resource to define the cases admitted to the COVID area. This screening method was used to confirm or rule out cases according to the Ministerial Resolution 193-2020; because diagnosis was based on epidemiological history and clinical characteristics of the patient. On the other hand, the laboratory for molecular tests was available since August 2020, before this date the tests were sent to other departments, complicating their use. In addition, some medical records had incomplete information of some of the variables. It is possible that there is insufficient statistical power for some associations. Regarding treatment, only the received drugs were considered, but not the doses. Finally, the results can only be extrapolated to similar populations.

Despite the limitations, this is one of the few studies that explores the relationship between saturation and adverse outcomes in high-altitude populations. In addition, our study identifies different factors that possibly influence the prognosis of the high-altitude resident with COVID-19.

In conclusion, in the Manuel Núñez Butron Regional Hospital, the risk factors for an adverse outcome (admission to the ICU or mortality) due to COVID-19 were male sex, age over 40 years, low saturation (< 80%) on admission, tachypnea (> 22 bpm) on admission, and diabetes.

The definition of admission for patients should be improved for future studies. On the other hand, it would be relevant to define temporal contexts because epidemiological surveillance in Peru determined the existence of two waves of COVID-19 cases; in addition, there are variants of the SARS-CoV-2 virus that appeared over time, as well as vaccines from different laboratories (Pfizer, Astrazeneca, Sinopharm). These variables could be included when replicating this study.

COVID-19 represents a huge public health burden globally and nationally, and identification of risk factors at hospital admission in high-altitude populations could help to take early measures that contribute to clinical improvement of the patients. These findings should be confirmed in future studies.
