# Supplementary material for: Risk factors for intensive care unit admission or mortality in adults hospitalized for COVID-19 at high altitude
Source: Rev Peru Med Exp Salud Publica. 2022 Jun 30;39(2):143–51. doi: 10.17843/rpmesp.2022.392.10721 (PMC11397585; doi:10.17843/rpmesp.2022.392.10721)
Supplement: Translation

# Translation: Factores de riesgo de ingreso a unidad de cuidados intensivos o mortalidad en adultos hospitalizados por COVID-19 en altura

**Authors:** Niza Alva, Giovana Asqui, Germán F. Alvarado, Francisco Muchica

**Affiliations:** 1 Programa de Medicina, Universidad Peruana de Ciencias Aplicadas, Lima, Perú.; 2 Hospital Regional Manuel Núñez Butron, Puno, Peú.

**Keywords:** COVID-19, Mortalidad, Hospitalizado, Altitud, Oximetría

## Abstract

**Objetivo.:**

Determinar los factores de riesgo de ingreso a unidad de cuidados intensivos (UCI) o mortalidad en pacientes hospitalizados por COVID-19 en un hospital de Puno, Perú.

**Materiales y métodos.:**

Estudio de cohorte retrospectivo en adultos hospitalizados entre abril y diciembre del 2020. Se evaluaron características sociodemográficas, funciones vitales, comorbilidades, tratamiento recibido y su asociación con el ingreso a UCI o mortalidad (desenlace adverso). Se utilizó regresión de Poisson con varianza robusta para calcular riesgos relativos (RR) crudos y ajustados con sus intervalos de confianza al 95% (IC95%).

**Resultados.:**

Se analizaron 348 historias clínicas. La mediana de edad en años fue 42,5 (RIC: 30,0; 58,0); el 38,2% fueron varones, y el 35,3% falleció o ingresó a UCI. Los que ingresaron con una saturación de oxígeno ≤ 75%, tuvieron 2,79 veces la probabilidad de tener el desenlace adverso (p < 0,001), en comparación con los que ingresaron con una saturación ≥ 85%; los que ingresaron con un valor entre 75-79% tuvieron 2,92 veces la probabilidad de tener el desenlace adverso (p < 0,001); asimismo, los que ingresaron con saturación entre 80-84% tuvieron 1,70 veces la probabilidad de presentar el desenlace adverso; sin embargo la diferencia no fue estadísticamente significativa (p=0,066). Además, hubo mayor riesgo de desenlace adverso en pacientes de sexo masculino RR= 1,75 (p<0,001), edad > 40 años RR 3,5 (p=0,001), taquipnea RR=1,66 (p=0,010), o con diabetes, RR = 1,53 (p=0,011).

**Conclusiones:**

. Los factores de riesgo para ingresar a UCI o mortalidad por COVID-19 fueron el sexo masculino, edad mayor de 40 años, saturación baja, diabetes y taquipnea.

## INTRODUCCIÓN

Un importante número de casos de neumonía grave se notificó en Wuhan, China en diciembre de 2019 ^1^. Posteriormente, se identificaría a esta nueva enfermedad denominada COVID-19, causada por el virus del síndrome respiratorio agudo severo tipo 2 (SARS-CoV-2) ^1^. Debido a su extensa propagación mundial, el 11 de marzo de 2020 la Organización Mundial de la Salud (OMS), proclamó a la COVID-19 como una pandemia, sugiriendo a los países seguir una estrategia integral dirigida a prevenir infecciones y muertes ^2^. Hasta el 26 de octubre del 2021, existieron más de 4,9 millones de muertes en el mundo provocadas por esta enfermedad. En respuesta, el Ministerio de Salud (MINSA) de Perú, decretó el estado de emergencia sanitaria nacional ^3^ ^).^ Los casos confirmados por coronavirus en el país ascendieron a 2 197 052, con 200 118 fallecidos y con una letalidad del 9,11% ^4^. Para agosto del 2020, Perú llegó a ser el país con mayor mortalidad por la COVID-19, por número de habitantes ^4^ ^,^ ^5^. 

Existen estudios que postulan que la prevalencia e impacto de la COVID-19 podrían verse disminuidos por un potencial factor protector como la altura ^6^ ^,^ ^7^; ya sea por los cambios drásticos que suelen existir en estos entornos, elevada radiación ultravioleta y sequedad del aire ^6^. Asimismo, la disminuida expresión de la enzima convertidora de angiotensina (ECA) en el endotelio pulmonar de los habitantes de altura podría generar un factor protector fisiológico al ser el lugar de unión del SARS-CoV-1 y SARS-CoV-2 ^6^ ^,^ ^7^. Sin embargo, se tratan de estudios ecológicos, con afirmaciones no concluyentes, por lo que resulta necesario más investigación. 

Existen diversos factores asociados a la mortalidad por esta enfermedad, que pueden agruparse en características sociodemográficas, funciones vitales, comorbilidades, exámenes auxiliares, tiempo de enfermedad y tratamiento recibido ^3^. Dentro de las características sociodemográficas, se destaca el sexo masculino y una edad mayor a 60 años ^3^ ^,^ ^8^ ^-^ ^10^. En el grupo de comorbilidades, resalta la obesidad (IMC > 30) ^3^ ^,^ ^11^ ^,^ ^12^, diabetes *mellitus* ^11^ ^-^ ^14^ e hipertensión arterial (HTA) ^3^ ^,^ ^11^ ^,^ ^15^. Estas características predisponen a cualquier individuo a un mayor riesgo de infección por SARS-CoV-2 o a presentar una enfermedad más grave. 

La saturación de oxígeno (SatO2) es un importante factor pronóstico, ya que tener un bajo porcentaje en el ingreso hospitalario se asocia con mayor mortalidad ^3^ ^,^ ^15^ ^,^ ^16^. Un estudio realizado en Lima, ciudad a 161 m de altitud, encontró que tener una saturación < 90% al momento de la admisión se asociaba con un mayor riesgo de muerte en comparación con quienes llegaron con > 90% ^3^. No hemos encontrado estudios que determinen qué porcentaje de saturación indique un mal pronóstico en residentes de altura. Sin embargo, se sabe que los habitantes de altura tienen valores de SatO2 diferentes a los residentes a nivel del mar. Un estudio realizado en pobladores sanos de Huánuco (1894 m de altitud) y Cerro de Pasco (3399 m de altitud) encontró que la media de saturación fue 96,2 y 87,0%, respectivamente, por lo que concluyen que mientras mayor sea la altitud de residencia, los cambios en los valores normales de SatO2 son más notorios ^17^, cabe resaltar que este estudio fue realizado fuera del contexto de pandemia.

Puno es una región que se encuentra a una altitud de 3827 m, por lo que los resultados del presente estudio pueden ser de utilidad para entender la dinámica de la enfermedad en poblaciones de altura y para proponer futuros estudios.

El objetivo de esta investigación fue determinar los factores de riesgo de ingreso a la unidad de cuidados intensivos (UCI) o a mortalidad en una población de altura de adultos hospitalizados por la COVID-19 en un hospital de Puno, Perú.

**MENSAJE CLAVE**

Motivación para realizar el estudio: los factores de riesgo de desenlace adverso por COVID-19 no están bien definidos en poblaciones con altitudes mayores a 3000 m.

Principales hallazgos: los factores asociados a la muerte por COVID-19 fueron edad > 40 años, sexo masculino, diabetes, ingresar con una saturación de oxígeno menor a 80% y una frecuencia respiratoria > 22 rpm.

Implicancias: identificar estos factores de riesgo en una población situada a 3827 m de altitud, permitirá reconocer a los pacientes con peor pronóstico para implementar medidas pertinentes.

## MATERIALES Y MÉTODOS

### Diseño y contexto

Se diseñó un estudio de cohorte retrospectivo en el Hospital Regional Manuel Núñez Butrón (HRMNB) de Puno, Perú, que incluyó pacientes adultos hospitalizados en el área COVID entre abril y diciembre del 2020. El HRMNB es de categoría II, administrado por el Ministerio de Salud (MINSA) ubicado a una altitud de 3827 m. Los primeros casos por COVID-19 se registraron a inicios de abril dándose la primera ola entre los meses de julio y agosto.

### Población

Se incluyeron pacientes adultos (≥18 años), hospitalizados en áreas COVID del HRMNB con una residencia previa en Puno, mínima de dos meses. Se excluyeron pacientes que se retiraron voluntariamente y en los que finalmente no se confirmó el diagnóstico con la prueba rápida.

### Muestra

Se realizó un censo de los pacientes hospitalizados en área COVID del HRMNB de Puno de abril a diciembre del 2020, obteniendo 542 historias clínicas. Para definir un tamaño de muestra mínimo se utilizó Epidat 4.2. En base a un estudio ^3^ se construyó una tabla de contingencia y se calculó la probabilidad de morir entre expuestos y no expuestos, se consideró una razón de 0,33 con un nivel de confianza de 99,9%, potencia de 99,9%. La p1 representa la probabilidad de morir en pacientes con saturación baja (<85%) siendo esta de 61,6%; mientras que; la p2, es la probabilidad de morir en pacientes sin saturación baja (≥ 85%) que fue de 14%. El tamaño mínimo requerido para la muestra fue de 192 y considerando un 10% de historias mal llenadas, esta fue de 214.

### Variables

La variable dependiente fue el desenlace adverso, que estuvo compuesto por el ingreso a la UCI o mortalidad hospitalaria. Esta variable fue categórica dicotómica (falleció o ingresó a UCI / no falleció, ni ingresó a UCI). 

Las variables independientes fueron la edad en años (<40 / 40-59 / ≥60), sexo (femenino / masculino), tiempo de enfermedad (días), saturación de oxígeno al ingreso (≤75% / 76% - 79% / 81% - 84% / ≥85%), frecuencia respiratoria (taquipnea > 22 rpm / normal), entre otras funciones vitales. Las comorbilidades tuvieron respuesta dicotómica (sí / no) e incluyen diabetes *mellitus*, HTA, enfermedad renal crónica, insuficiencia cardiaca congestiva y obesidad. Respecto a los exámenes de laboratorio se resaltan hemoglobina (g/dL) y glucosa (mg/dL) como variables numéricas, además de linfopenia (≤1400 U/mm3 / >1400 U/mm3) y tipo de sangre (O+, A+, B+). Los medicamentos recibidos durante la hospitalización tuvieron respuesta dicotómica (sí/no) e incluyeron dexametasona, azitromicina, hidroxicloroquina e ivermectina, entre otros. Cabe resaltar que las funciones vitales y los valores de exámenes de laboratorio considerados, fueron los del ingreso hospitalario.

### Recolección de datos

La recolección de datos se llevó a cabo luego de la aprobación del Comité de Ética en Investigación de la Universidad Peruana de Ciencias Aplicadas y la autorización de la Dirección Regional de Salud (DIRESA) de Puno. Se obtuvo acceso a las historias clínicas de pacientes hospitalizados por la COVID-19 del HRMNB de Puno entre abril y diciembre de 2020. Se seleccionaron aquellas que cumplían con los criterios de selección. Entre junio y julio de 2021, se llenó una ficha de recolección de datos con las variables elegidas por revisión bibliográfica, posteriormente los datos se organizaron en Excel.

### Análisis de datos

Se usó el programa de análisis estadístico STATA v16. Para el análisis univariado, se calcularon porcentajes para las variables categóricas, medidas de tendencia central y de dispersión para las variables cuantitativas, se calculó el promedio y desviación estándar (DE) si hubo normalidad, de lo contrario, mediana y rango intercuartil (RIC). Para el análisis bivariado, se utilizaron las pruebas de T de Student / U de Mann Whitney y chi cuadrado / Fisher, según cumplimiento de supuestos. Por último, para el análisis de múltiples variables, se utilizó la regresión de Poisson con varianza robusta para calcular el riesgo relativo (RR) crudo y ajustado con intervalos de confianza del 95% (IC95%). Se consideró un alfa de 0,05. Las variables ingresaron al modelo según criterio epidemiológico / teórico. Se evaluó la colinealidad con el FIV (factor de inflación de la varianza) utilizando un punto de corte de 2.

### Aspectos éticos

El protocolo contó con la aprobación del Comité de Ética de la Universidad Peruana de Ciencias Aplicadas (SCEI 024-01-22 PI 496-20) y con la autorización de la DIRESA de Puno. Dado que los datos ya figuraban en las historias clínicas, el equipo de investigación no tuvo contacto directo con los sujetos de investigación y se guardó confidencialidad. Código de registro PRISA: EI00001585. 

## RESULTADOS

Se obtuvieron un total de 542 historias clínicas de pacientes hospitalizados por la COVID-19 en el hospital de Puno, de abril a diciembre de 2020. De estas, fueron excluidas 194 por no cumplir con criterios de inclusión o por no encontrarse disponibles. Finalmente, se consideraron 348 como parte de la muestra analizada (Figura 1).

**Figura 1:** Flujograma del proceso de selección de historias clínicas de pacientes hospitalizados por COVID-19 en un hospital de Puno, Perú.

### Análisis descriptivo

Con respecto a las características básicas (Tabla 1), el 48,2% tenía menos de 40 años, el 29,1% entre 40 y 59 años y el 22,7% tenía 60 años o más; el 61,8% fueron mujeres. Con referencia al desenlace, el 18,4% ingresó a UCI, mientras que el 5,5% del total usó ventilación mecánica invasiva y la mortalidad hospitalaria fue del 29,9%. En comorbilidades, el 13,8% tenía diabetes, 7,5% tenía HTA, un 4,6% tenía obesidad y, por último, el 21,8% tenía una comorbilidad y el 6,4% tenía dos o más de ellas.

**Tabla 1:** Características de los pacientes hospitalizados en áreas COVID. Hospital Manuel Núñez Butrón. Puno, Perú 2020.

Variables	n (%)
Características básicas	
Edad (años)	
<40	168 (48,2)
40 - 59	101 (29,1)
≥ 60	79 (22,7)
Sexo	
Masculino	133 (38,2)
Femenino	215 (61,8)
Motivo de ingreso	
COVID	157 (45,1)
Otro	191 (54,9)
Desenlace	
Ingreso a UCI	64 (18,4)
Ventilación mecánica	19 (5,5)
Mortalidad hospitalaria	104 (29,9)
Estancia hospitalaria (días) ^a^	3,8 (1;5)
Comorbilidades	
Diabetes	48 (13,8)
HTA	26 (7,5)
Asma	1 (0,3)
Tuberculosis	1 (0,3)
Cáncer	1 (0,3)
ERC	5 (1,4)
ICC	6 (1,7)
EPOC	4 (1,2)
Enfermedad pulmonar intersticial	2 (0,6)
VIH	1 (0,3)
Obesidad	16 (4,6)
Número de comorbilidades	
0	247 (71,8)
1	75 (21,8)
≥ 2	22 (6,4)
Funciones vitales	
Saturación de oxígeno (%)	
≥ 85	242 (69,9)
80 - 84	19 (5,5)
76 - 79	21 (6,1)
≤ 75	64 (18,5)
Temperatura (°C) ^a^	36,6 (36,2;36,8)
Frecuencia respiratoria (rpm)	
Normal	163 (48,7)
Taquipnea	172 (51,3)
Frecuencia cardiaca (lpm) ^a^	89,9 (78;100)
Presión arterial sistólica (mmHg) ^a^	117,1 (105;128)
Presión arterial diastólica (mmHg) ^a^	74,5 (66;83)
Diagnóstico	
Diagnóstico clínico	168 (48,3)
Diagnóstico radiológico	
Positivo	77 (22,6)
Negativo	5 (1,6)
No se realizó	250 (75,8)
Prueba rápida	
Prueba rápida IgM positivo	233 (66,9)
Prueba rápida IgG positivo	285 (81,9)
Prueba molecular	
Positivo	14 (4,2)
Negativo	0 (0,0)
No se realizó	321 (95,8)
Manejo	
Anticoagulantes	
Dosis plena	51 (14,6)
Dosis profiláctica	57 (16,4)
No recibió	240 (69,0)
Ventilación no invasiva	
Cánula binasal	75 (21,6)
Máscara de reservorio	73 (29,9)
Medicamentos recibidos	
Dexametasona	130 (37,4)
Metilprednisolona	2 (0,6)
Prednisona	1 (0,3)
Acetaminofén	20 (5,8)
Ibuprofeno	5 (1,4)
Metamizol	151 (43,4)
Tramadol	49 (14,1)
Azitromicina	81 (23,3)
Meropenem	28 (8,1)
Imipenem	41 (11,8)
Vancomicina	96 (27,6)
Cefepime	1 (0,3)
Ceftriaxona	102 (29,3)
Cefazolina	7 (2)
Ampicilina	26 (7,3)
Amoxicilina	7 (2,01)
Clindamicina	30 (8,62)
Gentamicina	23 (6,6)
Ciprofloxacino	12 (3,5)
Metronidazol	5 (1,4)
Hidroxicloroquina	83 (23,9)
Ivermectina	78 (22,4)
IECAS	20 (5,8)
Vasopresores	14 (4)
Omeprazol	152 (43,7)
Salbutamol	69 (19,8)
Bromuro de ipratropio	68 (19,5)

a mediana (rango intercuartílico)

Algunos valores podrían tener distintos totales debido a datos faltantes.

UCI: unidad de cuidados intensivos. HTA: hipertensión arterial. ERC: enfermedad renal crónica. ICC: insuficiencia cardiaca congestiva. EPOC: enfermedad pulmonar obstructiva crónica. VIH: virus de inmunodeficiencia adquirida. TGO: transaminasa glutámica oxalacética. TGP: transaminasa glutámico pirúvica. PCR: proteína C reactiva. LDH: lactato deshidrogenasa.

Con relación a la saturación de oxígeno, el 69,9% de pacientes ingresó con una saturación mayor de 85%, el 5,5% ingresó con una saturación que oscilaba entre 80 y 84%, el 6,1% con una saturación que oscilaba entre 76 y 79% y un 18,5% ingresó con menos de 75% de saturación.

Respecto al diagnóstico, el 48,3% tuvo diagnóstico clínico. Además, un 66,9% dio positivo a la prueba rápida IgM y el 81,9% dio positivo a la prueba rápida IgG. En cuanto a los análisis de laboratorio, el promedio de hemoglobina fue de 15,1 g/dL con una desviación estándar (DE) de 3,0. Además, el 46,7% presentaba linfopenia y un 33,9% presentaba leucocitosis. La mediana de glucosa fue 102 mg/dL (RIC: 84,1;124,0). Con respecto al manejo, en ventilación no invasiva; es decir, soporte ventilatorio sin intubación endotraqueal, el 29,9% recibió máscara de reservorio; entre tanto, el 21,6% de los pacientes recibió cánula binasal. Los pacientes que recibieron dexametasona fueron 37,4%. Por otra parte, el 23,3% recibió azitromicina el 23,9% tomó hidroxicloroquina y el 22,4% tomó ivermectina.

### Análisis bivariado

En el análisis bivariado, se observó una asociación estadísticamente significativa entre la edad y desenlace adverso (p < 0,001) al igual que con el sexo masculino (p < 0,001) y motivo de ingreso (p < 0,001) (Tabla 2).

**Tabla 2:** Factores asociados a ingreso a UCI o fallecimiento. Análisis bivariado. Hospital Manuel Núñez Butrón. Puno, Perú 2020.

Variables	Falleció o ingresó a UCI n (%)	No falleció ni ingresó a UCI n (%)	Valor de p
Características básicas			
Edad (años)^ a^			
< 40	9 (5,4)	159 (94,6)	
40 - 59	57 (56,4)	44 (43,6)	<0,001
≥ 60	57 (72,2)	22 (27,8)	
Sexo ^a^			
Masculino	88 (66,2)	45 (33,8)	<0,001
Femenino	35 (16,3)	180 (83,7)	
Motivo de ingreso ^a^			
Otro	18 (9,4)	173 (90,6)	<0,001
COVID	105 (66,9)	52 (33,1)	
Características hospitalarias			
Estancia hospitalaria (días) ^b^	4,4 (2,0;6,0)	3,4 (1,0;4,0)	0,022
Comorbilidades			
Diabetes ^a^			
No	86 (28,7)	214 (71,3)	<0,001
Sí	37 (77,1)	11 (22,9)	
HTA ^a^			
No	110 (34,2)	212 (65,8)	0,104
Sí	13 (50,0)	13 (50,0)	
ERC ^c^			
No	119 (34,7)	224 (65,3)	0,055
Sí	4 (80,0)	1 (20,0)	
ICC ^c^			
No	118 (34,5)	224 (65,5)	0,022
Sí	5 (83,3)	1 (16,7)	
Obesidad ^a^			
No	109 (32,8)	223 (67,2)	<0,001
Sí	14 (87,5)	2 (12,5)	
Funciones vitales			
Saturación (%)			
≥ 85	29 (11,9)	213 (88,1)	
80 - 84	11 (57,9)	8 (42,1)	<0,001
75 - 79	19 (90,5)	2 (9,5)	
≤ 75	62 (96,9)	3 (3,1)	
Frecuencia respiratoria ^a^			
Normal	17 (10,4)	146 (89,6)	<0,001
Taquipnea	98 (57,0)	74 (43,0)	
PAS (mmHg) ^b^	111 (95,0;130,0)	120 (109,0;127,0)	0,017
PAD (mmHg) ^b^	73 (60,0;81,0)	75 (69,5;83,0)	0,025
Manejo			
Anticoagulantes ^a^			
Dosis plena	38 (74,5)	13 (25,5)	<0,001
Dosis profiláctica	35 (61,4)	22 (38,6)	
No	50 (20,8)	190 (79,2)	
Dexametasona ^a^			
No	40 (18,4)	178 (81,6)	<0,001
Sí	83 (73,8)	47 (36,2)	
Azitromicina ^a^			
No	68 (25,5)	199 (74,5)	<0,001
Sí	55 (67,9)	26 (32,1)	
Hidroxicloroquina ^a^			
No	67 (25,3)	198 (74,7)	<0,001
Sí	56 (67,5)	27 (32,5)	
Ivermectina ^a^			
No	70 (25,9)	200 (77,1)	<0,001
Sí	53 (67,9)	25 (32,1)	

a Prueba de chi cuadrado.

b Mediana (rango intercuartil) Prueba U de Mann Whitney

c Prueba exacta de Fisher

HTA: hipertensión arterial. ERC: enfermedad renal crónica. ICC: insuficiencia cardiaca congestiva. PAS: presión arterial sistólica. PAD: presión arterial diastólica

En cuanto a las comorbilidades, la diabetes (p < 0,001) y obesidad (p < 0,001) tuvieron una asociación significativa con el desenlace adverso, no se observó asociación significativa con HTA (p=0,104).

Respecto a los signos vitales, se encontró asociación significativa entre la saturación de oxígeno y el desenlace adverso (p < 0,001), de igual manera con la frecuencia respiratoria (p < 0,001), menor presión arterial sistólica (p = 0,017) y menor presión arterial diastólica (p = 0,025).

Con respecto a los exámenes de laboratorio, se encontró asociación significativa entre el desenlace adverso y mayor nivel de hemoglobina (p < 0,001), así como con mayor nivel de glucosa (p < 0,001) y linfopenia (p < 0,001). Sin embargo, no se encontró asociación significativa entre el desenlace adverso y el grupo sanguíneo (p = 0,200).

Respecto a los fármacos utilizados en el manejo, se encontró asociación significativa entre el desenlace adverso y dexametasona (p < 0,001), azitromicina (p < 0,001), hidroxicloroquina (p < 0,001) e ivermectina (p < 0,001).

### Análisis de múltiples variables

En el análisis de múltiples variables se observó una asociación estadísticamente significativa entre la edad y el desenlace adverso, donde las personas entre 40 y 59 años tuvieron 3,7 veces la probabilidad de desarrollar un desenlace adverso (p < 0,001) y los mayores de 60 años tuvieron 3,5 veces el riesgo en comparación con quienes tenían menos de 40 años (p = 0,001) (Tabla 3). Asimismo, los varones tuvieron 1,8 veces el riesgo de desenlace adverso (p < 0,001) luego de ajustar por el resto de las variables en la ecuación. Respecto a las comorbilidades, los pacientes con diabetes tuvieron 1,3 veces el riesgo de desarrollar el desenlace adverso (p = 0,011) y los obesos, tuvieron 1,2 veces el riesgo de desarrollarlo, sin embargo, la asociación no fue significativa (p = 0,260). Por otro lado, tampoco se obtuvo una asociación estadísticamente significativa con la HTA (p = 0,851). Los pacientes con una saturación de oxígeno que osciló entre 80 y 84% tuvieron 1,7 veces el riesgo de desarrollar un desenlace adverso frente a quienes ingresaron con más de 85%, sin una asociación significativa (p = 0,066); en cambio, quienes tuvieron valores entre 75 y 79% tuvieron 2,9 veces la probabilidad de desarrollar un desenlace adverso (p < 0,001), lo mismo para quienes ingresaron con menos de 75%, quienes tuvieron un riesgo de 2,8 (p < 0,001).

**Tabla 3:** Factores asociados a ingreso a UCI o fallecimiento. Análisis de múltiples variables. Hospital Manuel Núñez Butrón. Puno, Perú 2020.

Variables	RRc	IC95%	Valor de p	RRa	IC95%	Valor de p
Edad (años)						
< 40	Referencia			Referencia		
40 - 59	10,53	5,44;20,36	<0,001	3,71	1,82;7,57	<0,001
≥ 60	13,46	7,02;25,82	<0,001	3,53	1,73;7,21	0,001
Sexo						
Femenino	Referencia			Referencia		
Masculino	4,06	2,93;5,63	<0,001	1,75	1,30;2,35	<0,001
Diabetes						
No	Referencia			Referencia		
Sí	2,68	2,12;3,40	<0,001	1,34	1,06;1,69	0,011
HTA						
No	Referencia			Referencia		
Sí	1,46	0,96;2,21	0,071	0,96	0,67;1,38	0,851
ERC						
No	Referencia					
Sí	2,30	1,45;3,66	<0,001	-	-	-
ICC						
No	Referencia					
Sí	2,41	1,64;3,55	<0,001	-	-	-
Obesidad						
No	Referencia			Referencia		
Sí	2,66	2,09;3,39	<0,001	1,21	0,86;1,72	0,260
Saturación						
≥ 85	Referencia			Referencia		
80 - 84	4,83	2,88;8,07	<0,001	1,70	0,96;3,00	0,066
75 - 79	7,61	5,29;10,97	<0,001	2,92	1,99;4,30	<0,001
≤ 75	8,08	5,72;11,41	<0,001	2,79	1,89;4,11	<0,001
Frecuencia respiratoria						
Normal	Referencia			Referencia		
Taquipnea (>22 rpm)	5,46	3,41;8,73	<0,001	1,66	1,12;2,46	0,010
Anticoagulantes						
No	Referencia			Referencia		
Dosis plena	3,57	2,66;4,80	<0,001	1,25	0,91;1,72	0,157
Dosis profiláctica	2,94	2,13;4,06	<0,001	1,04	0,80;1,36	0,730
Dexametasona						
No	Referencia			Referencia		
Sí	3,47	2,55;4,73	<0,001	1,04	0,77;1,41	0,769
Azitromicina						
No	Referencia			Referencia		
Sí	2,66	2,06;3,43	<0,001	1,05	0,79;1,40	0,705
Hidroxicloroquina						
No	Referencia			Referencia		
Sí	2,63	2,06;3,44	<0,001	0,90	0,73;1,12	0,386
Ivermectina						
No	Referencia					
Sí	2,62	2,03;3,37	<0,001	-	-	-

RRc: riesgo relativo crudo, RRa: riesgo relativo ajustado, HTA: hipertensión arterial, ERC: enfermedad renal crónica, ICC: insuficiencia cardíaca congestiva.

Los pacientes que ingresaron con taquipnea (> 22 rpm) tuvieron el 1,7 veces el riesgo de un desenlace adverso, con una diferencia estadísticamente significativa (p = 0,010). No hubo asociación entre el uso de anticoagulantes y el riesgo de tener un desenlace adverso (p > 0,05) De la misma manera sucedió entre el desenlace adverso y dexametasona (p = 0,769), azitromicina (p = 0,705) e hidroxicloroquina (p = 0,386). Las variables «enfermedad renal crónica» e «insuficiencia cardiaca congestiva» fueron eliminadas del modelo ajustado debido a un importante porcentaje de valores perdidos y la variable «ivermectina» se eliminó por colinealidad con hidroxicloroquina.

## DISCUSIÓN

Los factores de riesgo identificados para ingresar a UCI o fallecer fueron la edad mayor de 40 años, sexo masculino, ingresar con una saturación de oxígeno menor a 80%, ingresar con una frecuencia respiratoria > 22, o tener diabetes *mellitus*. No se encontró una asociación significativa entre el desenlace adverso con la obesidad o HTA. Como hallazgos secundarios, el 18,4% de pacientes ingresó a UCI y el 29,9% falleció. El 30,1% ingresó con una saturación < 85%. La mortalidad hallada en este estudio fue menor (29,9%) en comparación con lo reportado en otros hospitales, 49% ^3^ y 46,4% ^18^; sin embargo, estos estudios no serían comparables al ser de diferentes contextos, características y momentos de la pandemia en el país.

A pesar de ello, en el presente estudio se reportan características y factores de riesgo similares a las descritas en otras cohortes ^3^ ^,^ ^13^ ^,^ ^15^ ^,^ ^19^ ^-^ ^21^. Para empezar, se observó asociación entre una edad mayor de 40 años y el desenlace adverso, lo que también se puede evidenciar en otros estudios ^9^ ^,^ ^10^ ^,^ ^13^ ^,^ ^23^, esto posiblemente se deba al aumento en la prevalencia de enfermedades crónicas en este grupo de edad. Asimismo, podría deberse a la vulnerabilidad frente a infecciones, explicada por la inmunosenescencia ^9^.

En el presente estudio se observó una asociación entre el sexo masculino y el desenlace. Esta asociación ha sido descrita en estudios realizados en ciudades a nivel del mar ^3^ ^,^ ^8^ ^,^ ^10^. Se ha descrito que mecanismos biológicos como una respuesta inmune disminuida de los hombres frente al de las mujeres podrían estar involucrados ^23^. Se sugiere también que las hormonas sexuales como los andrógenos, podrían tener un papel importante al regular proteínas como la proteasa transmembrana de serina 2 (TMPRSS2), esta proteína participa en la entrada y propagación viral, lo que se traduce en formas de COVID-19 más severas ^24^.

Se identificó como factor de riesgo el tener una saturación de oxígeno de ingreso < 80%, valor que difiere del punto de corte planteado en otros estudios similares realizados en poblaciones a nivel del mar ^3^ ^,^ ^15^ ^,^ ^18^ y en poblaciones con una menor altitud que Puno ^19^. Esto se podría explicar por la baja presión de oxígeno, ya que la saturación descrita en pacientes de altura sin ninguna patología es menor, comparada con la de residentes a nivel del mar ^17^, por lo que se esperaría que en pacientes con la COVID-19, la saturación sea aún menor ya que es una patología que agrega hipoxemia. Es difícil establecer un punto de corte en poblaciones de altura y se describe una relación inversamente proporcional entre la saturación de oxígeno y el nivel de altura ^17^; hacen falta más estudios para cuantificar esta relación y determinar los puntos de corte.

Asimismo, se encontró una asociación significativa entre taquipnea (>22 rpm) y el desenlace adverso. Esta relación es comprensible debido a que aumentar la frecuencia respiratoria es la respuesta fisiológica a la hipoxemia y la acidosis ^25^. Nuevamente, es necesario destacar que se necesitan más estudios que evalúen la taquipnea en condiciones de altura para determinar los puntos de corte que indicarían un mal pronóstico en esta población.

Respecto a las comorbilidades, se encontró asociación entre el desenlace adverso y la diabetes *mellitus* (DM), como en otros estudios ^11^ ^-^ ^14^ ya que la DM involucra una inmunodepresión por alteraciones de la respuesta inmune que incluyen una mayor producción de citocinas proinflamatorias, que agregan inflamación al paciente con la COVID-19 ^26^. No obstante, no se encontró asociación entre el desenlace adverso y la obesidad, como sí sucedió en otros estudios ^20^ ^,^ ^22^ donde el desenlace fue mortalidad y, al igual que la DM, la obesidad involucra un estado proinflamatorio debido a citocinas (TNF-alfa, MCP-1 e IL-6) producidas por el tejido adiposo ^12^. Por otro lado, si bien estudios reportan asociación entre la mortalidad y la HTA ^3^ ^,^ ^11^ ^,^ ^15^, no se encontró asociación entre esta y desenlace adverso en el análisis ajustado. Se postula que existe una mayor expresión de la ECA en hipertensos debido al tratamiento con inhibidores de la ECA, esto podría aumentar el riesgo de formas severas de la COVID-19 ^14^.

En los resultados de laboratorio, no se encontró asociaciones estadísticamente significativas; sin embargo, existe literatura que describe que, en residentes de gran altitud se ha encontrado disminución en los niveles de plaquetas, en pacientes graves.

Por último, no se encontró asociación significativa entre el desenlace adverso y el recibir anticoagulantes (enoxaparina) ya sea en dosis profiláctica (40 mg) o plena (60 mg), tampoco con dexametasona, azitromicina o hidroxicloroquina. Respecto a la hidroxicloroquina, existen estudios que demuestran que no existe beneficio en disminuir la mortalidad, comparado con quienes no la reciben ^27^ ^,^ ^28^. Por el contrario, existe literatura que respalda la asociación entre el uso de dexametasona en dosis bajas y una reducción de la mortalidad, en pacientes con COVID-19 severo ^29^.

La limitación principal de este estudio es el uso de la prueba rápida, debido a su baja sensibilidad diagnóstica. Sin embargo, resultó ser el recurso más accesible para definir los casos que ingresaron al área COVID. Este método de tamizaje fue usado para confirmar o descartar casos según la Resolución Ministerial 193-2020; ya que, el diagnóstico se basaba en antecedentes epidemiológicos y características clínicas del paciente. Por otra parte, el laboratorio para el uso de pruebas moleculares estuvo disponible desde agosto del 2020, antes de este mes las pruebas se enviaban a otros departamentos, lo que complicaba su uso. Además, algunas historias clínicas presentaron un registro incompleto para algunas de las variables. Es posible que no exista un poder estadístico suficiente para algunas asociaciones. En cuanto al tratamiento, solo se consideraron los medicamentos recibidos, más no las dosis. Por último, los resultados se podrán extrapolar solo a poblaciones similares.

A pesar de las limitaciones, este es uno de los pocos estudios que explora la relación entre la saturación y los desenlaces adversos en poblaciones de altura. Además, identifica diferentes factores que posiblemente influyen en el pronóstico del residente de altura con la COVID-19.

En conclusión, en el Hospital Regional Manuel Núñez Butron los factores de riesgo para un desenlace adverso (ingreso a UCI o mortalidad) por la COVID-19 fueron el sexo masculino, edad mayor de 40 años, saturación baja (< 80%) al ingreso hospitalario, taquipnea (> 22 rpm) al ingreso hospitalario y tener diabetes.

Para estudios futuros, debe mejorarse la definición de ingreso para los pacientes. Por otra parte, sería relevante delimitar contextos temporales debido a que la vigilancia epidemiológica en Perú determinó la existencia de dos olas de casos de COVID-19; además, existen variantes del virus SARS-CoV-2 que fueron incorporándose en el transcurso del tiempo, así como también se usaron vacunas de diferentes laboratorios (Pfizer, Astrazeneca, Sinopharm). Estas variables podrían incluirse al replicar un estudio similar a este.

La COVID-19 supone una enorme carga para la salud pública a nivel mundial y nacional por lo que la identificación de factores de riesgo al ingreso hospitalario en poblaciones a gran altitud podría ayudar para tomar medidas precoces que contribuyan a la mejoría clínica de cada paciente. Estos hallazgos deben de confirmarse en estudios futuros.
